# Visualized Efficacy of Andexanet Alfa in an Elderly Trauma Patient with an Unstable Pelvic Fracture

**DOI:** 10.31662/jmaj.2024-0007

**Published:** 2024-06-03

**Authors:** Junya Tsurukiri, Takeo Nagura, Takashi Kanemura, Kenji Isaka, Hidefumi Sano

**Affiliations:** 1 Department of Emergency and Critical Care Medicine, Tokyo Medical University Hachioji Medical Center, Tokyo, Japan

**Keywords:** trauma, andexanet alfa, hemorrhagic shock

An 89-year-old man taking rivaroxaban, an oral, direct Factor Xa inhibitor, for arterial fibrillation was referred to our department with hemorrhagic shock after falling accidentally. Pelvic images revealed a pelvic fracture classified as LC-III according to the Young-Burgess classification with active bleeding in the pelvic muscles (Figure 1). Therefore, an intravenous injection of 400 mg was administered at a rate of 30 mg/min, followed by an infusion of 4 mg/min of andexnet alfa for 2 h. After 15 min of starting injection, emergency angiography revealed the disappearance of bleeding (Figure 2). Embolization was discontinued, and 1,960 mL of erythrocytes and 1,680 mL of fresh frozen plasma were administered within 24 h. Although andexanet alfa can reduce antifactor Xa activity during administration, its role in trauma patients has not been sufficiently studied. Given its specific mechanism of action, andexanet alfa may be advantageous for improving hemostatic performance in major trauma patients ^(1), (2), (3)^.

**Figure 1.**
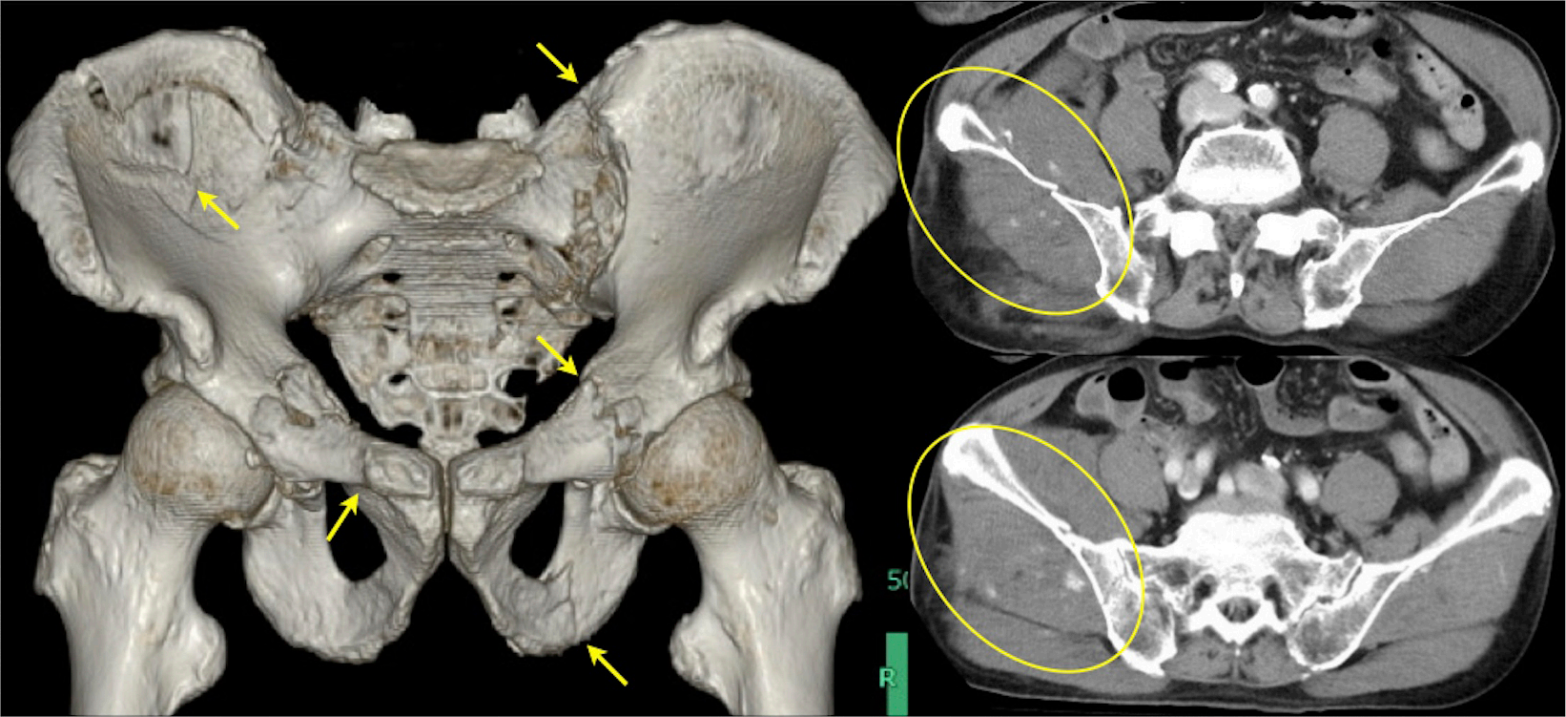
Computed tomography (CT) of the pelvis revealed a pelvic fracture classified as LC-III according to the Young-Burgess classification (arrow). Contrast-enhanced CT also revealed contrast medium extravasation in the pelvic muscles (circle).

**Figure 2.**
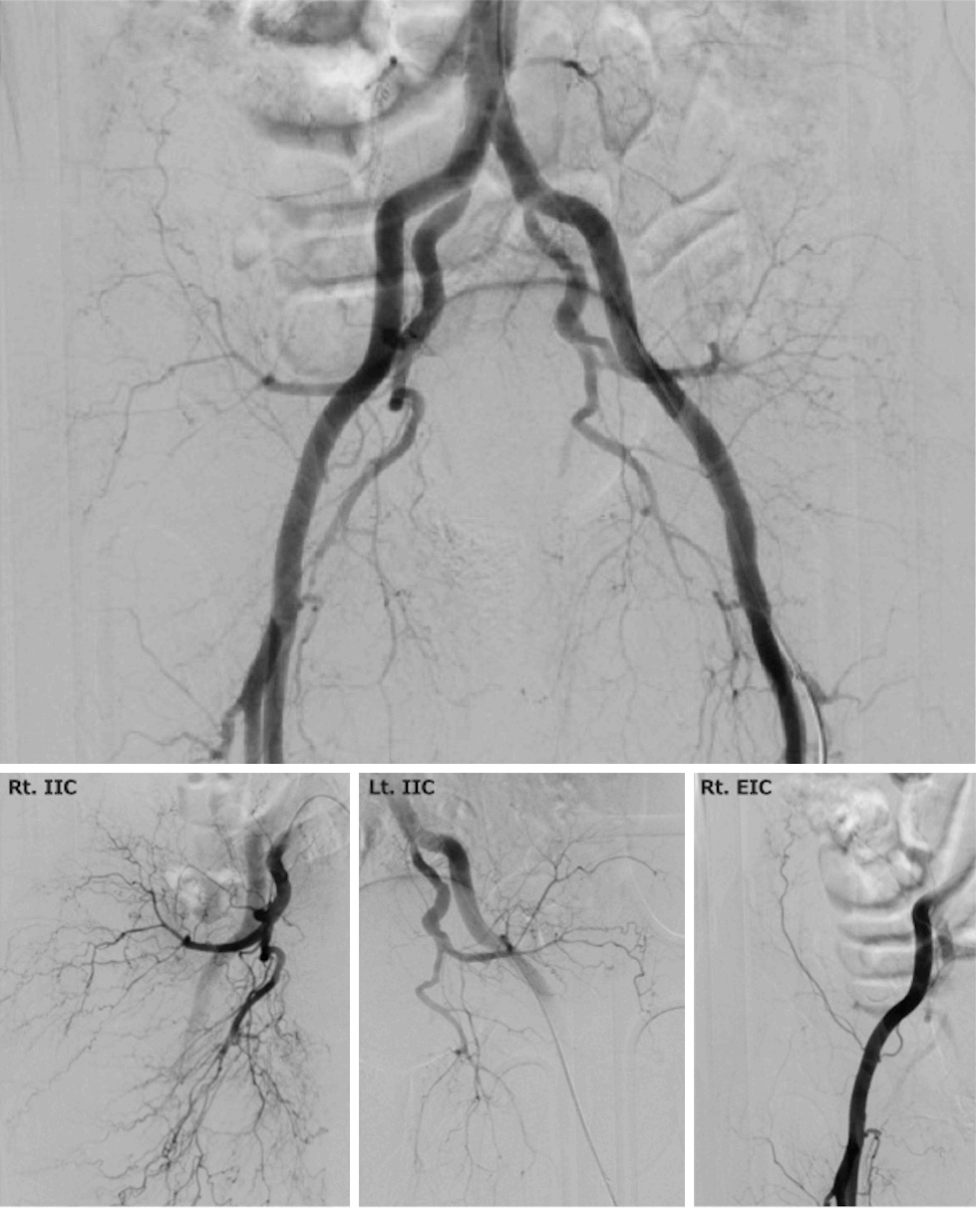
Emergency angiography of the pelvis revealed the disappearance of contrast medium extravasation. Selective angiography revealed that the bleeding was halted. Rt. IIC: right internal iliac artery, Lt. IIC: left internal iliac artery, Rt. EIC: right external iliac artery.

## Article Information

### Conflicts of Interest

None

### Acknowledgement

The authors would like to thank Enago (www.enago.jp) for the English language review.

### Author Contributions

Conceived and designed the experiments: TJ

Contributed to the interpretation of data: NT, KT, and IK

Approved the final version to be submitted: SH

### Informed Consent

Written informed consent was obtained from the patient for the publication of this case report and any accompanying images.
